# Functional Characterization of the Human BRCA1 ∆11 Splicing Isoforms in Yeast

**DOI:** 10.3390/ijms25147511

**Published:** 2024-07-09

**Authors:** Alvaro Galli, Francesca Bellè, Arcangelo Fargnoli, Maria Adelaide Caligo, Tiziana Cervelli

**Affiliations:** 1 Yeast Genetics and Genomics, Laboratory of Functional Genetics and Genomics, Institute of Clinical Physiology, National Research Council, 56124 Pisa, Italy; alvaro.galli@cnr.it (A.G.); f.belle01@gmail.com (F.B.); arcangelo.fargnoli@hotmail.it (A.F.); 2 Molecular Genetics Unit, Department of Oncology, University Hospital of Pisa, 56126 Pisa, Italy; m.caligo@ao-pisa.toscana.it

**Keywords:** BRCA1 ∆11 splicing isoforms, yeast-based functional assay, BRCA1 intronic variants, BRCA1 localization

## Abstract

*BRCA1*, a crucial tumor suppressor gene, has several splicing isoforms, including Δ9–11, Δ11, and Δ11q, which lack exon 11, coding for significant portions of the protein. These isoforms are naturally present in both normal and cancerous cells, exhibiting altered activity compared to the full-length BRCA1. Despite this, the impact on cancer risk of the germline intronic variants promoting the exclusive expression of these Δ11 isoforms remains uncertain. Consequently, they are classified as variants of uncertain significance (VUS), posing challenges for traditional genetic classification methods due to their rarity and complexity. Our research utilizes a yeast-based functional assay, previously validated for assessing missense BRCA1 variants, to compare the activity of the Δ11 splicing isoforms with known pathogenic missense variants. This approach allows us to elucidate the functional implications of these isoforms and determine whether their exclusive expression could contribute to increased cancer risk. By doing so, we aim to provide insights into the pathogenic potential of intronic VUS-generating BRCA1 splicing isoforms and improve the classification of BRCA1 variants.

## 1. Introduction

**Figure 1:**
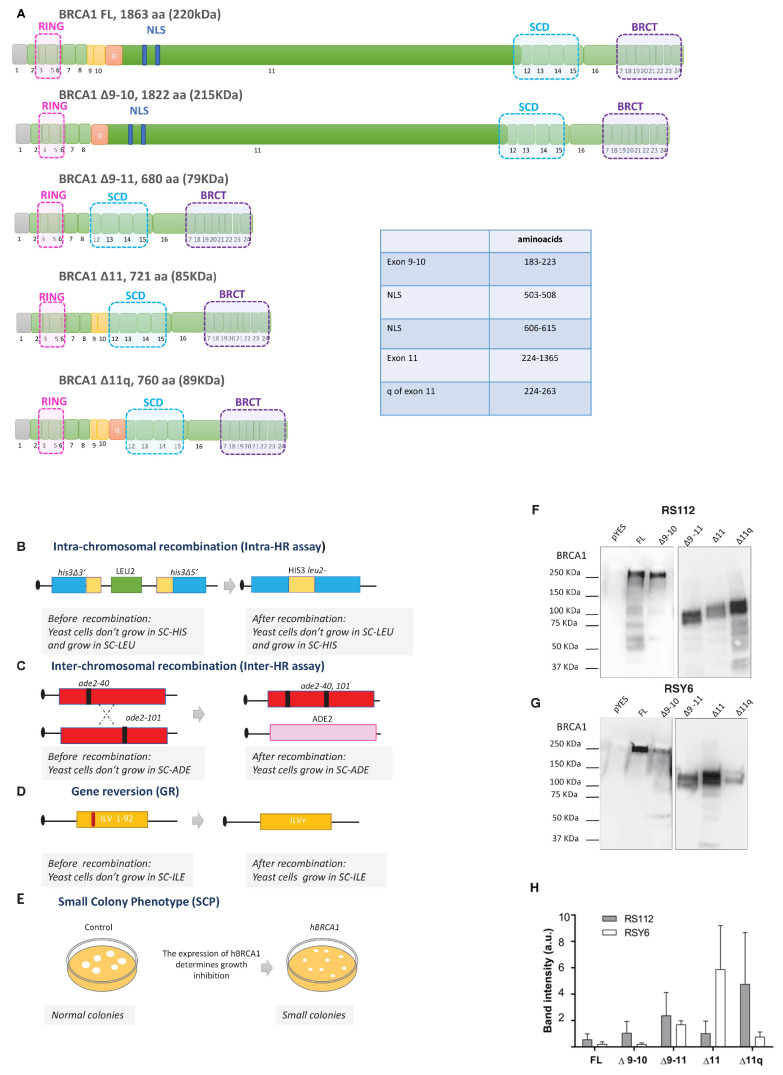
(**A**) Schematic representation of *BRCA1* gene. It consists of 23 exons coding for a protein of 1863 aa. Exon 1 is indicated in gray because it is a non-coding region. Exon 4 is missing due to an initial omission during BRCA1 protein characterization. The 11 is the longest exon. (**B**–**D**). Schematic representation of intra-chromosomal, inter-chromosomal recombination (HR), and Gene reversion (GR) substrates. (**B**) Intra–HR substrate consists of two his3 alleles deleted at 3′ and 5′ end, separated by the *LEU2* gene. Recombinants are scored as the number of colonies grown in synthetic medium without histidine (SC-HIS). (**C**) Interchromosomal HR consists of two alleles of the ade2 gene carrying two different mutations located in distinct chromosomes. Recombinants are scored as the number of colonies grown in synthetic medium without adenine (SC-ADE). (**D**) GR substrate consists of the mutant ilv1-92 gene. Revertants are scored as colonies grown in synthetic medium without isoleucine (SC-ILE). (**E**) The readout of the small colony phenotype assay (SCP) is the number of cells composing a single colony. (**F**,**G**) Western blot of proteins extracted from RS112 (**F**) and RSY6 (**G**) strains to determine the expression of BRCA1 isoforms. Representative Western blots (top) and band quantification (**H**) are shown. Band intensity values reported in the graph are divided by 10^8^. Bars show mean ± SEM.

Germline mutations of the DNA repair gene *BRCA1* have been reported to increase the lifetime risk of hereditary breast and ovarian cancer (HBOC) [1,2]. The full-length BRCA1 protein (FL) is composed of 1863 amino acids forming a 220 kDa protein characterized by three well-conserved functional domains: the RING domain (exons 2–7), a region encoded by exons 11–13, and the BRCT domain (exons 16–24). The RING domain functions as an E3 ubiquitin ligase. The BRCT domain is a phosphoprotein-binding domain with specificity for proteins phosphorylated by ATM/ATR kinases. Exon 11 is one of the biggest human exons, spanning 3426 bases and encoding for more than 60% of the total protein (1142 aa) [3]. It contains two nuclear localization signals (NLSs) and the protein-binding domains for proteins involved in DNA damage repair (Figure 1A) [4]. Interestingly, it has been proposed as a “coldspot”, namely a region that can tolerate variations because missense variants localized in this exon are classified as benign [5]. However, many missense variants in this region are classified as variants of uncertain significance (VUS) and likely pathogenic by recent classification methods [6,7].

Alternative or aberrant splicing events produce the protein isoforms ∆9–10, ∆11, and ∆11q that contain residual exon 11 sequences, and ∆9–11 that also lacks exons 9 and 10. The BRCA1 ∆9–10 isoform has been found in several negative controls and is not distinguishable from the FL [8]. BRCA1 ∆11 isoforms are involved in either cell death or proliferation, depending on their expression level and exclusivity. For instance, cells expressing only BRCA1 ∆11 show DNA double-strand break (DSB) repair defects [9]. A role for BRCA1 ∆11 splicing isoforms has been identified in the response to PARPi treatment. The poly (ADP-ribose) polymerase-1 (*PARP1*) gene encodes for a nuclear protein that attaches a poly-ADP-ribose (PAR) polymer (PARylation) to itself and to other target proteins. Inhibition of PARP1 in cancer therapy is a recently adopted strategy to treat cancers where DNA repair is defective, such as HBOC caused predominantly by pathogenic variants in *BRCA1* or *BRCA2* [10]. In the long term, treatment with PARPi can become ineffective due to the development of PARPi resistance. One mechanism that cancer cells use to develop PARPi resistance is by promoting the expression of the BRCA1 ∆11q isoform. This isoform retains some BRCA1 activity compared to the truncating mutation, allowing cells to survive PARPi treatment [11,12]. By contrast, in *BRCA1* wild-type (WT) tumors, exon 11 skipping increases sensitivity to PARPi, suggesting that these splicing isoforms are as functionally active as the protein WT [13]. This might be due to the different cellular localization of BRCA1 ∆11 isoforms compared to the WT protein, but the mechanism by which BRCA1 localization influences cancer development and therapy response remains unknown [14,15,16].

The BRCA1 intronic variants c.4096+1G>A (IVS11+1) and c.4096+3A>G (IVS11+3) determine the increase in expression of alternative splicing isoforms BRCA1 ∆11 and ∆11q, respectively [8,17]. Their classification, necessary for genetic and clinical counseling, is still conflicting [18,19]. Classification of rare variants, such as IVS11+1 and IVS11+3, may result in difficulty with classical genetic approaches requiring the identification of many individuals with each variant. Functional assays (FAs) are considered by the American College of Medical Genetics (ACMG) guidelines as strong evidence to infer pathogenicity or neutrality even for rare variants [20]. Yeast has been largely used to develop FAs for cancer-associated genes [21,22,23]. The Small Colony Phenotype (SCP) assay is basically the first test used to assess the functional impact of BRCA1 missense variants [24,25]. Expression of BRCA1 WT in yeast determines the formation of colonies containing a lower number of cells than colonies formed by yeast expressing truncated BRCA1 proteins or pathogenic variants [25]. We previously reported that the expression of BRCA1 cancer-associated variants increases homologous recombination (HR) and gene reversion (GR) in yeast, confirming the potentiality of this genetic model in cancer biology [7,26,27]. For instance, the pathogenic variant p.C39Y, located in the RING domain, causing a defect in the DSB repair in human cells, determines HR and GR alteration in yeast. By contrast, the benign variant p.D67Y does not affect HR and GR [7,28,29]. We validated these three yeast-based assays for missense variants in the coding sequence and demonstrated that by combining their results with a computational approach, we can predict the impact of these variants on BRCA1 activity [7]. In the present paper, we used these yeast-based FAs to evaluate the functional activity of BRCA1 ∆11 isoforms.

## 2. Results

### 2.1. BRCA1 Δ11 Isoforms Have a Functional Impact in Yeast Functional Assays

To express BRCA1 Δ11, Δ9–11, and Δ11q, we constructed plasmids based on pYES2 by assembling PCR fragments in yeast (Figure S1). The assembling has been performed so that the cDNA is expressed under the control of the inducible promoter GAL1 as described in the Material and Methods Section (Supplementary Figure S1). The three splicing variants together with the BRCA1 FL and BRCA1 Δ9–10 isoform (Figure 1A), considered as WT [8], were expressed in the yeast strains RS112 and RSY6, containing the substrates to measure HR and gene reversion (GR), respectively (Figure 1B–E). Specifically, the diploid strain RS112 was used to determine the ability of BRCA1 FL and BRCA1 isoforms to affect intra- and inter-chromosomal HR at *HIS3* and *ADE2* loci (Figure 1B,C) and the haploid RSY6 strain was used to determine the effect on GR, by scoring for revertants at the *ILV1* locus (Figure 1D). We previously demonstrated that BRCA1 missense pathogenic variants increase the frequency of HR and GR [26,27]. SCP assay was also performed for RS112 expressing BRCA1 isoforms. The readout of the assay is the inhibition of growth determined by BRCA1 WT, not observable when pathogenic BRCA1 variants are expressed (Figure 1E).

**Figure 2:**
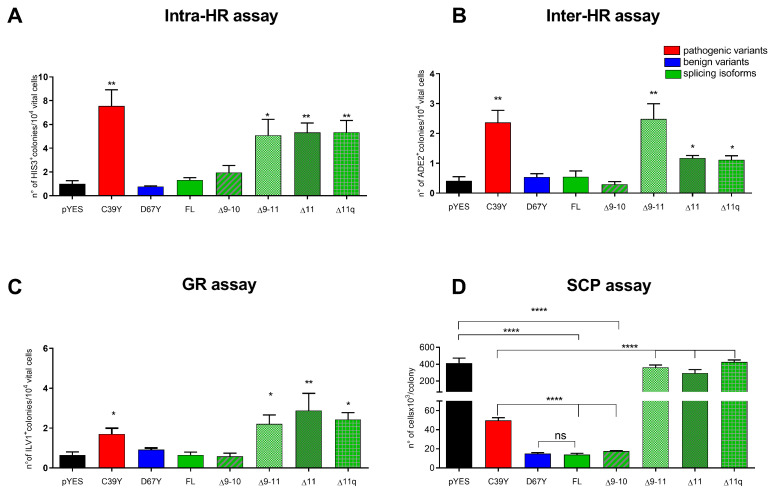
Functional assays in yeast for BRCA1 Δ11 isoforms. The assays presented have been validated for missense variants in the coding sequence. The assays have been performed overexpressing the BRCA1 coding sequence in yeast. The pathogenic variant is shown in red, benign in blue, and BRCA1 Δ11 isoforms in green. (**A**) Spontaneous intra-homologous recombination (intra-HR), (**B**) inter-homologous recombination (inter-HR) assay, (**C**) reversion assay (GR), and (**D**) SCP assay have been evaluated. The graph represents the mean ± SD of 3–5 independent experiments. Statistical analyses of graphs (**A**–**C**) were performed with unpaired Student’s *t*-test comparing all data with BRCA1 FL. Statistical analyses of graph (**D**) were performed using unpaired and one-way Anova. All the analyses were performed with GraphPad Prism 8, GraphPad Software Inc. (Boston, MA, USA). Values of *p* < 0.05 were considered statistically significant (* *p* < 0.05, ** *p* < 0.01, **** *p* < 0.0001). Bars show mean ± SEM.

The expression of the BRCA1 proteins was evaluated by Western blot of whole cell lysate from cells grown in 5% galactose for 24 h at 30 °C. BRCA1 splicing isoforms are expressed in both strains, RSY6 and RS112 (Figure 1F–H). The BRCA1 ∆9–11, BRCA1 ∆11, and ∆11q isoforms induced a statistically significant increase in both intra- and inter-chromosomal HR as compared to BRCA1 FL and BRCA1 Δ9–10 (Figure 2A,B). For comparison, results obtained and recently published for the BRCA1 pathogenic p.C39Y and the benign p.D67Y variants have been considered [7]. Pathogenic missense variant p.C39Y increased HR at both loci whereas expression of the benign variant p.D67Y did not affect HR under the same conditions (Figure 2A,B). The expression of the BRCA Δ9–11, Δ11, and Δ11q isoforms also induced a statistically significant increase in GR as compared to BRCA1 FL and BRCA1 Δ9–10. BRCA1 FL and BRCA1 Δ9–10 show a frequency of GR comparable to cells containing the empty plasmid (Figure 2C). The expression of the pathogenic p.C39Y variant also increased GR while the benign variant p.D67Y did not, as already reported [7]. Finally, the effect of BRCA1 Δ9–11, Δ11, and Δ11q isoforms on the SCP in the diploid strain RS112 has been analyzed. The expression of BRCA1 Δ9–11, Δ11, and Δ11q determined the formation of colonies with a significantly higher number of cells as compared to BRCA1 FL and the Δ9–10. This increase was even higher than that induced by the pathogenic variant p.C39Y (Figure 2D), which did not reach the level observed in cells transformed with the empty plasmid. Notably, the expression of BRCA1 FL and BRCA1 Δ9–10 allowed the formation of colonies with a very low number of cells as compared to the control (pYES2) (Figure 2D).

In general, we can conclude that results obtained in these FAs indicate that BRCA1 Δ9–11, Δ11, and Δ11q isoforms show a loss of function comparable to BRCA1 pathogenic missense variants.

### 2.2. BRCA1 Δ11 Isoforms Have a Cytoplasmic Localization

**Figure 3:**
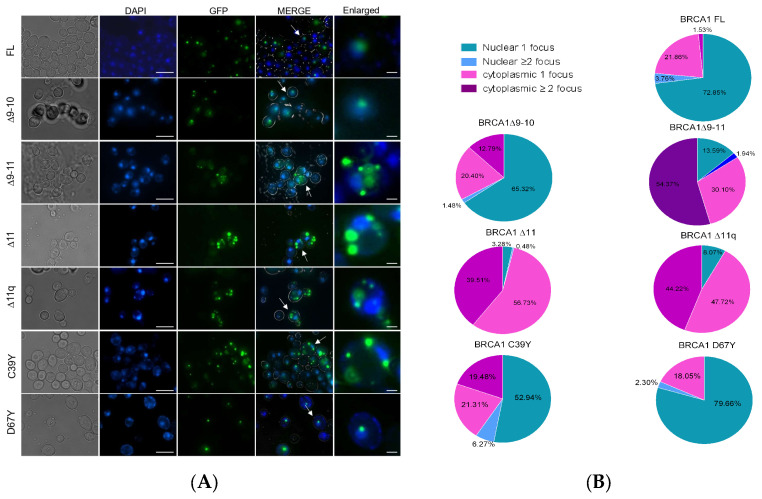
Localization of the BRCA1 Δ11 isoforms. (**A**) To study the localization of BRCA1 Δ11 isoforms, the plasmids with the BRCA1 isoforms fused to GFP at the carboxy-terminal were transformed in yeast. Nuclei were stained with DAPI. Representative fluorescence microscopy images of the localization of BRCA1 Δ11 isoforms and two missense variants are shown. The most noticeable difference between FL, Δ9–11, Δ11, and Δ11q is that FL forms one focus that co-localized with nuclear DNA and/or one cytoplasmic focus, and the variants form more than one focus, localized mainly in the cytoplasm as it occurs for pathogenic variant p.C39Y. Scale bar: 5 µm and 1 µm for the enlarged picture on the right of panel (**A**). (**B**) quantification of BRCA1 foci localization in the nucleus and in the cytoplasm.

It has been previously shown that in yeast, BRCA1 FL forms one nuclear focus and that BRCA1 pathogenic missense variants tend to localize into the cytoplasm as observed in mammalian cells [30]. To study the impact of deletion of exon 11 on BRCA1 localization, we constructed a pYES2-based vector expressing BRCA1 FL, Δ9–10, Δ9–11, Δ11, and Δ11q isoforms fused to GFP at the C-ter by transformation-associated recombination (TAR) (see Section 4.1, Supplementary Figure S1). At the same time, as control, we produced the plasmids for the expression of BRCA1 FL, ∆9–10, the pathogenic p.C39Y, and the benign variant p.D67Y fused to GFP. BRCA1 FL and BRCA1 Δ9–10 localized preferentially as a single nuclear focus (Figure 3A). In fact, 72.85% and 65.35% of cells with BRCA1 FL and ∆9–10, respectively, have a single nuclear inclusion (Figure 3B). In most cells (almost 80%), BRCA1 Δ9–11, Δ11, and Δ11q variants localized in the cytoplasm, as a single or multiple focus. However, a variable proportion of cells show multiple cytoplasmic foci (Figure 3A,B). Similarly, the pathogenic p.C39Y induces an increase in cytoplasmic localization of BRCA1 in comparison to BRCA1 FL and Δ9–10. Meanwhile, the benign p.D67Y variant shows the same localization pattern as BRCA1 FL and Δ9–10 (Figure 3A,B).

## 3. Discussion

Yeast has been used to develop FAs to classify missense variants of genes that can be involved in or associated with human diseases [22,23,31]. Yeast-based assays have been exploited to investigate the function of BRCA1 exonic missense variants, but never to evaluate BRCA1 splicing isoforms. The *BRCA1* IVS11+1 and IVS11+3 variants generate alternative splicing leading to the formation of BRCA1 ∆11 and ∆11q isoforms, respectively [8,17,32]. These variants have been classified by ClinVar (https://www.ncbi.nlm.nih.gov/clinvar/, accessed on 7 June 2024) and BRCA exchange database (https://brcaexchange.org/, accessed on 7 June 2024) as VUS. Basically, it is uncertain whether the defective activity of BRCA1 Δ11 splicing isoforms has a role in cancer predisposition. Splicing isoforms such as ∆9–10, ∆9–11, ∆11, and ∆11q are ‘naturally occurring’ splicing isoforms, produced by wild-type alleles in non-malignant tissues [8]. Incidentally, an increase in exon 11 skipping has been found to allow survival of patients carrying homozygous nonsense BRCA1 pathogenic variants [33]. However, the IVS11+3 variant has been reported as likely pathogenic because it co-segregates with breast and ovarian cancer [18] and as likely benign because it was identified in a healthy homozygous carrier [34]. In addition, the other intronic variant IVS11+1, has been identified in several patients [35,36] and is a founder variant in Italy [19].

Recently, we have validated four FAs and developed a method, called yBRCA1, in yeast that reliably assesses the functional impact of BRCA1 missense variants. The yBRCA1 method, which combines the results of the four yeast-based FAs, resulted in accuracy, sensitivity, and specificity of over 95% [7]. Nevertheless, the yBRCA1 method was validated for missense variants. In the present paper, we used yBRCA1 to evaluate the pathogenicity of the splicing isoforms ∆9–10, ∆9–11, ∆11, and ∆11q. We expressed the BRCA1 isoforms in two yeast strains and determined their effect on intra- and inter-chromosomal HR, GR, and SCP assay. The results clearly indicated that the deletion of exon 9–10 did not affect BRCA1 activity. By contrast, the deletion of exon 11 confers an impairment in the functional activity of BRCA1. Our findings indicate that the expression of BRCA1 variants ∆9–11, ∆11, and ∆11q leads to an increase in HR, GR, and SCP, as observed with pathogenic variants. For instance, we previously showed that the p.Y1703C variant, initially classified as a variant of uncertain significance (VUS), altered all four FAs. As a result, according to the outcomes of yBRCA1, we may classify *BRCA1* variants leading to ∆11 splicing isoforms as pathogenic [7]. This observation is in agreement with in vitro functional studies on BRCA1 ∆11 splicing isoforms which have highlighted defective activity in DNA repair (Table 1).

Besides conducting the yeast-based FAs, we also examined the localization of the BRCA1 splicing isoforms. The localization of BRCA1 is a crucial aspect of its biology, as studies have shown that cytoplasmic localization is associated with a loss of tumor suppressor activity and an increase in the invasiveness of cancer cells [16]. Exon 11 is important for BRCA1 localization because, in addition to multiple protein-binding sites, including those recognized by the RAD51 and RAD50 complex, it contains two NLSs [4]. However, the two canonical NLSs are insufficient for predominant nuclear localization that occurs only when BARD1 (BRCA1 associated RING domain protein 1) form a heterodimer with BRCA1 binding the RING domain. As a result, BARD1 masks the NH2-terminal nuclear export signal that promotes cytoplasmic localization. Therefore, BRCA1 nuclear localization requires NLSs and the RING domain [37]. BARD1 binding to the RING domain is crucial not only for the localization of BRCA1, but also for the DSB repair and E3 ubiquitin ligase activities [4,38]. In human and mouse cells, BRCA1 Δ11 isoforms partially localize in the nucleus and can form nuclear foci after DNA damage (Table 1). This suggests that the deletion of exon 11 does not determine a complete loss of function. In the present paper, we observed that, in yeast, BRCA1 ∆11 isoforms localize in the cytoplasm in around 80–90% of cells and in the nucleus in 3–15% of cells. By contrast, BRCA1 FL and Δ9–10 mostly localize in the nucleus. This observation aligns with findings obtained in both mouse and human cells and suggests that these proteins can diffuse through the nuclear pore [39]. Similarly, it has been previously reported that BRCA1 missense pathogenic variants predominantly localize in the cytoplasm in yeast [30].

At the moment, the mechanism by which the BRCA1 Δ11 isoforms induce HR and GR in yeast is unknown. BRCA1 has a role in protecting cells from reactive oxygen species (ROS) that can be produced by oxidative metabolism [40]. Antioxidant genes have been identified as transcriptional targets of BRCA1, indicating that BRCA1 protects cells against oxidative stress [41,42]. Increased ROS levels can induce DNA damage and thus activate DNA damage response [43]. In our system, the higher background of HR and GR observed in cells expressing the BRCA1 Δ11 isoforms is not determined by the increase in ROS (Figure S2). Moreover, the induction of HR and GR is not dependent on the level of the BRCA1 proteins, since BRCA1 Δ11 isoforms show different protein levels in our yeast strains though the fold increase in HR, GR, and SCP is comparable. Our results support the findings that BRCA1 Δ11 isoforms have a residual activity that can lead to cell transformation. It has been shown that changes in the balance between BRCA1 FL [18,44] and the naturally occurring BRCA1 Δ11 isoforms may predispose to breast and ovarian cancer and promote resistance to PARPi [11,12]. Most doubts regarding the pathogenicity of variants leading to BRCA1 Δ11 isoforms arise from the observation of a healthy homozygous carrier of the c.4096+3A>G variant [34] and the fact that mice BRCA1 ∆11/∆11 have a less severe phenotype compared to BRCA1 FL null mice [45]. However, in 2019, Zong et al. demonstrated that the loss of 53BP1 rescued BRCA1 ∆11/∆11 lethality, suggesting that the pathogenicity of the BRCA1 Δ11 isoforms may be strictly dependent on the genetic background [46].

**Table 1:** In vitro functional studies on BRCA1 ∆11 splicing isoforms.

Cell Lines	Method of Expression	Activity	Localization	PARPi Sensitivity	Refs.
NIH 3T3, simian Cos-7 kidney	Plasmid transfection	n.d.	BRCA1 FL localizes in the nucleus; BRCA1∆11 localizes in the cytoplasm	n.d	[14]
293-EBNA (human kidney cell line), COS-7	Plasmid transfection	n.d.	BRCA1 FL localizes in the nucleus; BRCA1∆11q localizes in the cytoplasm	n.d	[15]
Bosc 23 cells (293T-based cell line)	Plasmid transfection.	n.d.	BRCA1 FL localizes in the nucleus; BRCA1∆11q localizes in the cytoplasm	n.d	[47]
Mammary cells from mice	Retrovirus infection	BRCA1 ∆11 cells form abnormal mammary tissue in mice		n.d	[48]
BRCA1^∆11/∆11^ MEF (Mouse Embryonic stem Fibroblast)		Reduced phosphorylation of BRCA1 11∆ and Rad51 foci formation after radiation (IR)	Murine BRCA 11∆ localizes mainly in the cytoplasm but it is present also in the nucleus.	n.d	[49]
SUM149PT (breast cancer cells)	CRISPR/Cas9 gene editing	Low level of IR-induced BRCA1 and RAD51 foci	n.d	PARPi and cisplatin resistance	[11,12]
MCF-7, MDA-MB-231 (breast cancer cells)	Oligo promoting exon 11 skipping	n.d	n.d	Increased PARPi sensitivity	[13]

## 4. Materials and Methods

### 4.1. Yeast Strains, DNA Transformation and Cloning

The BY4741 (MATa ura3Δ leu2Δ his3Δ met15Δ), the haploid RSY6 (MATa ura3-52 leu2-3, -112 trp5-27 arg4-3 ade2-40 ilv1-92 HIS3::pRS6), and its derivative diploid strain RS112 (MATa/MATα ura3-52/ura3-52 leu2-3,112/leu2-Δ98 trp5-27/TRP5 ade2-40/ade2-101 ilv1-92/ilv1-92 arg4-3/ARG4 his3Δ50-pRS6-his3Δ30/his3-Δ200 LYS2/lys2-801) of *Saccharomyces cerevisiae* were used. Complete (YPAD) and synthetic media lacking uracil (SC-URA), leucine (SC-LEU), adenine (SC-ADE), histidine (SC-HIS), and isoleucine (SC-ILE) were prepared according to the standard techniques [50].

Plasmids carrying the BRCA1 FL (1863 aa), the ∆9–10 (1822 aa), ∆11 (721 aa), ∆11q (760 aa) that contain residual exon 11 sequences, and ∆9–11 (680 aa) that also lacks exons 9 and 10 (Figure 1A), were constructed in yeast (BY4741 strain) by transformation-associated recombination (TAR), which allows for the assembly of yeast expression vectors by using PCR fragments sharing 60 base pairs of homology [51,52]. As described in Supplementary Figure S1, we synthesized DNA inserts with different terminal homologies by PCR with specific primers (Supplementary Table S1), designed to skip well-defined exonic regions. We transformed them into the BY4741 strain together with the linearized pYES2 plasmid. The plasmid pPT63 (Supplementary Table S2) was used as a template for the BRCA1 sequence, which contains the BRCA1 FL sequence (a kind gift of Gaël Millot) [24]. HR between the two inserts and the pYES2 backbone results in the creation of the BRCA1 inserted in the pYES2 plasmid downstream of the GAL1 promoter. To study the intracellular localization of these BRCA1 isoforms, we generated new plasmids that expressed BRCA1 isoforms fused to GFP by TAR as described before (Supplementary Table S2). The GFP sequence was amplified from the plasmid pDCLryEGFP with the primers described in Supplementary Table S1 (a kind gift of Dr Blake R. Peterson) [53]. These DNA fragments share terminal homology to fuse GFP to the BRCA1 3′ end. The plasmids for the expression of the p.C39Y and the p.D67Y BRCA1 variants were constructed by site-specific mutagenesis using primers recently reported [7].

Yeast strains were transformed with DNA by using the lithium acetate method with single-stranded DNA as a carrier, following the procedure described in [54]. Transformant colonies were selected in a solid medium lacking uracil (SC–URA). Colonies were grown for 4 days at 30 °C and further analyzed. Single clones were picked from the plates and plasmids were purified from yeast cells and amplified in *E. coli*. Plasmids were sequenced to confirm the accuracy of the constructs.

### 4.2. Protein Extraction and Western Blotting

The BRCA1 protein level was determined in yeast whole cell lysate (WCL) from RS112 and RSY6 strains expressing BRCA1 FL, BRCA1 Δ9–10, and BRCA1 Δ11 isoforms. Single colonies were initially grown in 10 mL of SC-URA glucose liquid medium for 24 h at 30 °C under constant shaking. Then, a cell pellet was washed twice in water and inoculated in 20 mL of SC-URA 5% galactose medium. Cells were incubated for 24 h at 30 °C under shaking. Thereafter, the cultures were pelleted and washed in water. WCL was performed as previously described [7]. WCLs, obtained from cell cultures with a comparable number of cells, were electrophoresed on a 4–10% SDS–polyacrylamide TGX pre-cast gel and transferred on nitrocellulose membrane by Trans-Blot^®^ Turbo™ Transfer System (Bio-Rad, Milan, Italy). Blots were imaged using the ChemiDoc MP Imaging system (Bio-Rad, Milan, Italy). BRCA1 was detected using an Anti-BRCA1 mouse antibody diluted 1:300 (clone MS110, Calbiochem, Merck, Milan, Italy). BRCA1 band intensity was adjusted for variation in the protein loading between lanes normalizing for the total amount of protein by the Image Lab 6.1 software (Bio-Rad, Milan, Italy).

### 4.3. Functional Assays

As previously described, the diploid strain RS112 allows measurement of intra-chromosomal HR events between two his3 alleles deleted at the 3′ and 5′ terminus, sharing 400 bp of homology. RS112 measures also inter-chromosomal HR events, as this diploid strain carries the ade2-40 and ade2-101 alleles. Specifically, an intra-chromosomal HR event leads to the restoration of the HIS3 gene, allowing cells to grow in SC-HIS medium, and an inter-chromosomal HR event leads to the restoration of the ADE2 gene and allows cells to form colonies in SC-ADE medium [55]. GR events can be measured in the haploid strain RSY6 because it contains the ilv1-92 allele and cannot grow in SC-ILE medium. A GR event allows cells to grow in SC-ILE [27].

To analyze whether the expression of BRCA1 FL, BRCA1 Δ9–10, and BRCA1 Δ11 isoforms affect intra- and inter-chromosomal HR, single colonies of RS112 strain carrying the BRCA1 expression plasmids were inoculated into 5 mL of SC-URA-LEU 2% glucose medium and incubated at 30 °C for 24 h. Aliquots corresponding to 10^7^ cells were then incubated in 5 mL SC-URA-LEU containing 5% galactose for 24 h at 30 °C under constant shaking. Then, cells were washed twice, counted, diluted, and plated as recently reported. The frequency of intra- and inter-chromosomal HR events was expressed as the number of HIS3 colonies/10^4^ total cells and number of ADE2 colonies/10^5^ total cells, respectively. The effect of these BRCA1 isoforms on GR was determined in the haploid RSY6 strain by inoculating single clones in glucose overnight, and then into galactose medium (5%) for 24 h to allow BRCA1 expression; thereafter, cells were plated. GR frequency was expressed as the total number of ILV1 colonies/10^6^ cells [7].

The small colony phenotype assay (SCP), based on the ability conferred by the expression of BRCA1 pathogenic variants to restore yeast growth, was performed with the RS112 strain as follows: single colonies of RS112 carrying BRCA1 isoforms were inoculated in SC-URA 2% glucose for 24–48 h under shaking; then, cells were washed and diluted. Aliquots corresponding to 150–250 cells were plated in SC-URA with glucose and with galactose. The effect on colony size is determined by directly counting the number of cells per colony. Single colonies were picked up from each plate, resuspended in 1 mL of water, and counted by a hemocytometer. The results were expressed as number of cells per colony [7,24,25].

### 4.4. BRCA1 Localization

RS112 strains transformed with the plasmids for the expression of BRCA1 isoforms fused to GFP were grown overnight in 5 mL of SC-URA with glucose at 30 °C. The day after, 0.5 mL was added to 4.5 mL of SC-URA with galactose and grown. After 2 h 30′, 6.25 µL of DAPI (5 µg/µL) was added to the culture that was let grown for an additional 1 h 30′. At the end of growth, cells were washed with 25 mL of water, resuspended in 1 mL of water, centrifuged, and then resuspended in 100 µL of water. Then, 10 µL of cell suspension was dropped on a slide and visualized with a Nikon ECLIPSE Ti2-E inverted fluorescence microscope (Nikon Europe, Stroombaan, The Netherlands) equipped with a 60× objective. The localization of BRCA1 isoforms was counted in 100 cells showing DAPI staining and GFP signal.

## 5. Conclusions

In summary, our yeast-based FAs indicate that the residual activity exhibited by splicing isoforms is insufficient to maintain protein activity at levels comparable to the FL isoform. Despite containing the main functional domains, these isoforms exhibit characteristics akin to pathogenic variants. Thus, our data suggest that genomic variants that lead to the expression of BRCA1 Δ11 isoforms, such as IVS11+1 and IVS11+3, strongly promote the pathogenicity of BRCA1.

## Data Availability

All data are present in the article and in Supplementary Materials.

## Supplementary Materials

The following supporting information can be downloaded at: https://www.mdpi.com/article/10.3390/ijms25147511/s1.
