# Supplementary material for: Functional Characterization of the Human BRCA1 ∆11 Splicing Isoforms in Yeast
Source: Int J Mol Sci. 2024 Jul 9;25(14):7511. doi: 10.3390/ijms25147511 (PMC11276823; doi:10.3390/ijms25147511)
Supplement: Supplementary file 1 [file ijms-25-07511-s001.zip › ijms-3084126-supplementary.pdf]

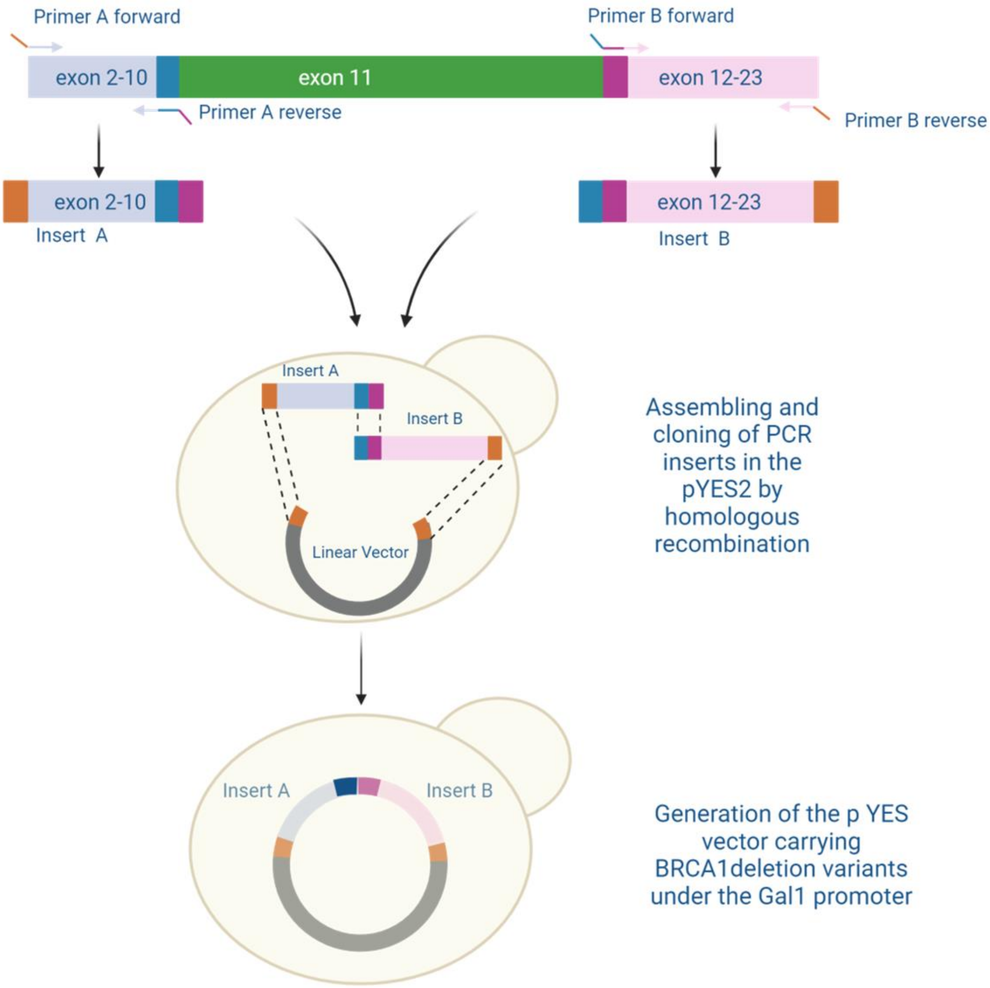

Type of inserts	Amplicon size	Primers' pairs
Insert A _Δ9-11	547 bp	BRCA1 forward HindIII/delta 9-11 rev
Insert A_Δ11	670 bp	BRCA1 forward HindIII /delta 11 rev
Insert A_Δ911q	787 bp	BRCA1 forward HindIII / delta 11q rev
Insert B_Δ9-11	1496 bp	delta 9-11 for/ BRCA1 reverse HindIII
Insert B_Δ11	1496 bp	delta 11 for/ BRCA1 reverse HindIII
Insert B_Δ911q	1496 bp	delta11q for/ BRCA1 reverse HindIII
Insert C_GFP	777 bp	yEGFP for/ yEGFP REV

Figure S1 Schematic representation of the strategy of plasmids' construction. To assemble BRCA1 carrying deletion of different exons, we performed PCR with specific primers designed in such a way to skip well-defined exonic regions and transformed it in yeast together with the linearized pYES2 vector. Specifically, the outer primers carried homology with the vector pYES2, and contained the restriction site *Hind*III; the internal primers carried a tail of 30 nt homologous with the adjacent fragment. Homologous recombination between the two inserts and then with the vector backbone results in the creation of the BRCA1 isoforms and cloning in the pYES2 vector. For the expression BRCA1 isoforms in frame with GFP at the C-ter, the assembling was performed with three inserts, A , B and C consisting in yGFP. The obtained plasmids werw purified from yeast cells and amplified in *E.coli*.

Table S1 list of primers used for the construction of plasmids

**Name**	**Sequence 5'→3'**	**Purpose**
BRCA1 forward HindIII	CTATAGGGAATATTAAGCTTATGGATTTATCTGCT CTTCG	Forward primer for PCR of insert A
BRCA1 reverse HindIII	CCGAGCTCGGTACCAAGCTTTCAGTAGTGGCTG TGGGGGA	Reverse primer for PCR of insert B
delta9-11 rev	TTTCACTCTCACACCCAGATGCTGCTTCACCCAA TTCAATGTAGACAGAC	Cloning by TAR, insert A
delta 11 rev	TTCACTCTCACACCCAGATGCTGCTTCACCCTTTT TTGCAGAATCCAAAC	Cloning by TAR, insert A
delta11q rev	TTTCACTCTCACACCCAGATGCTGCTTCACCCTG ATACTTTTCTGGATGC	Cloning by TAR, insert A
delta9-11 for	CTCAAAAGACGTCTGTCTACATTGAATTGGGTG AAGCAGCATCTGGGTGT	Cloning by TAR, insert B
delta 11 for	GATGAAATCAGTTTGGATTCTGCAAAAAAGGGT GAAGCAGCATCTGGGTG	Cloning by TAR, insert B
delta11q for	CAGCTGAGAGGCATCCAGAAAAGTATCAGGGT GAAGCAGCATCTGGGTGT	Cloning by TAR, insert B
yEGFP for	CTGATACCCCAGATCCCCCACAGCCACTACATGT CTAAAGGTGAAGAATT	Cloning GFP at 3'end by TAR, insert C
yEGFP REV	AGCACACTGGCGGCCGTTACTAGTGGATCCCTAT TTGTACAATTCATCCA	Cloning GFP at 3'end by TAR, insert C

TAR: transformation-associated recombination

Table S2 List of plasmids used in the paper

Plasmid	Size (bp)	description	source
pPT63	12,934	BRCA1 mCherry	[24]
pDCLryEGFP	7,668	Yeast optimized EGFP	[48]
YCpGAL1::BRCA1 (Δ9-10)		BRCA1 (Δ9-10)	[7]
pYES BRCA1 FL	11,458	BRCA1 FL	This study
pYES BRCA1 FL-GFP	12,152	BRCA1 FL-GFP	This study
pYES BRCA1 Δ9-11	7,908	BRCA1 Δ9-11	This study
pYES BRCA1 Δ9-11-yGFP	8,604	BRCA1 Δ9-11-yGFP	This study
pYES BRCA1 Δ11	8,028	BRCA1 Δ11	This study
pYES BRCA1 Δ11-yGFP	8,724	BRCA1 Δ11-yGFP	This study
pYES BRCA1 Δ11q	8,145	BRCA1 Δ11q	This study
pYES BRCA1 Δ11q-yGFP	8,841	BRCA1 Δ11q-yGFP	This study
pYES BRCA1 C39Y	11,458	BRCA1 with missense mutation, c.116G>A	[7]
pYES BRCA1 C39Y yGFP	12,152	BRCA1 with missense mutation_yGFP	This study
pYES BRCA1 D67Y	11,458	BRCA1 with missense mutation, c.199G>T	[7]
pYES BRCA1 D67Y yGFP	12,152	BRCA1 with missense mutation_yGFP	This study

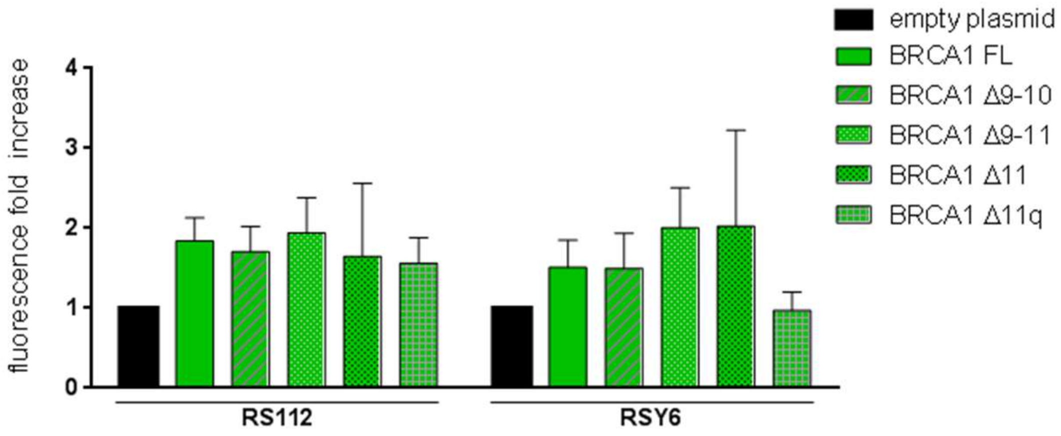

Figure S2 Total ROS quantification of yeast cells expressing BRCA1 isoforms. RSY6 and RS112 strains were grown in SC-URA+GLU O/N at 30°C under shaking conditions. The day after cells were diluted ten-fold in SC-URA+GAL and grown for 24h at 30°C to induce the expression of BRCA1 proteins. After 24h hours the cultures were diluted two-fold in SC-URA+GAL grown for other 2h in SC-URA+GAL. The intracellular ROS were measured by CellROX Deep Red Reagent (C10422, Thermo Fisher Scientific). According to the manufacturer's instructions, cells were incubated for 30 min with CellROX reagent, then washed three times with PBS. For each sample, 30.000 events were analyzed by CytoFLEX (Beckman coulter). The mean signal intensities of the fluorescence emission were compared between strains containing the empty plasmid and those expressing BRCA1 isoforms. ROS measurement was carried out in biological triplicates.
